# Reversible 3D optical data storage and information encryption in photo-modulated transparent glass medium

**DOI:** 10.1038/s41377-021-00581-y

**Published:** 2021-07-07

**Authors:** Zhen Hu, Xiongjian Huang, Zhengwen Yang, Jianbei Qiu, Zhiguo Song, Junying Zhang, Guoping Dong

**Affiliations:** 1 College of Materials Science and Engineering, Kunming University of Science and Technology, 650093 Kunming, China; 2 State Key Laboratory of Luminescent Materials and Devices, School of Materials Science and Engineering, South China University of Technology, 510640 Guangzhou, China; 3 School of Physics, Beihang University, 100191 Beijing, China

**Keywords:** Optical data storage, Lithography

## Abstract

Transparent glass has been identified as a vital medium for three-dimensional (3D) optical information storage and multi-level encryption. However, it has remained a challenge for directly writing 3D patterning inside a transparent glass using semiconductor blue laser instead of high-cost femtosecond laser. Here, we demonstrate that rare earth ions doped transparent glass can be used as 3D optical information storage and data encryption medium based on their reversible transmittance and photoluminescence manipulation. The color of tungsten phosphate glass doped with rare earth ions change reversibly from light yellow to blue upon alternating 473 nm laser illumination and temperature stimulation, resulting in the reversible luminescence modulation. The information data could be repeatedly written and erased in arbitrary 3D space of transparent glass, not only showing the ability of the excellent reproducibility and storage capacity, but also opening opportunities in information security. The present work expands the application fields of luminescent glass, and it is conducive to develop a novel 3D data storage and information encryption media.

## Abstract (web summary)

Three-dimensional (3D) optical information can be written in transparent glass using a focused 473 nm laser beam, which will be read by photo-modulation.

## Graphical abstract

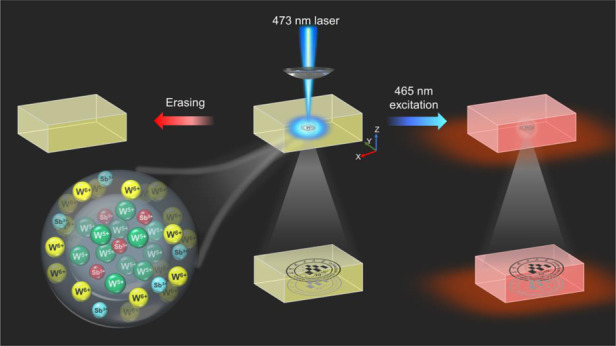

## Introduction

The advent of the information age is accompanied by the rapid growth of digital information, which is in urgent need for the development of huge storage space and security media. Optical storage technology with the huge storage capacity and low cost has become a new choice for information storage^1,2^. At present, the commercial optical storage devices mainly include Blu-ray discs, digital versatile discs, and compact discs^3–5^. However, due to the optical diffraction limitation, the optical storage technology faces a storage capacity bottleneck. To improve storage density, the traditional approach uses reducing laser wavelength or increasing numerical aperture of objective lens^6–9^. Recently, the super-resolution optical technology has been developed to break through the limit of optical diffraction^10–12^. Meanwhile, the three-dimensional (3D) information storage medium has been a good selection for improving the information storage capacity and extending storage life^13–17^. At present, the transparent glass has been proved as a viable information recording 3D medium under the processing of near-infrared femtosecond laser^14,15,18^. However, the femtosecond laser is a complicated and expensive equipment in contrast to the visible continuous semiconductor laser, and it usually requires a higher photon energy and a more complex operating process. In addition, the photostimulated luminescence particles or metal nanoparticles embedded in glasses were explored as an optical storage medium^19^. For the photostimulated luminescence particles based on the design of defect traps, it is relative difficult for the preparation of the photostimulated luminescence particles embedded glasses^20^. Thus these characteristics urge us to develop a new information recording technology for the bulk glass medium.

The application of rare earth doped materials can be extended based on their luminescence modification induced by the external field stimulation such as electric, magnetic and temperature fields^21–24^. Compared with the modification approaches of the photoluminescence of rare earth ions based on the external temperature, electric and magnetic fields, the external light field approach for photoluminescence modification of rare earth ions exhibits some advantages such as simple manipulation, real time, high efficiency, high safety and good repeatability. At present, the reversible photoluminescence modification of rare earth doped ceramics or films were demonstrated based on photochromic effect, exhibiting the potential application in some fields such as holographic memory and optical data storage^25–29^. However, 3D optical data storage cannot be achieved in the photo-control ceramics due to their opacity. Compared to the photochromic ceramics, the photochromic bulk glasses are easier to realize the 3D optical data storage due to their transparent property, which improves the capacity of data storage. Therefore, the development of photo-modulated glass is urgently needed to realize the 3D optical information storage applications.

**Fig. 1:**
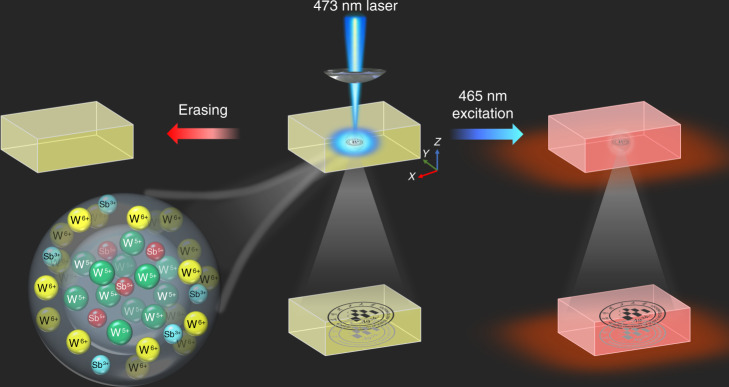
Schematic of the writing, reading, and erasing of optical information. A focused 473 nm laser beam is irradiated into the glass, and the blue area is the photochromic region of the glass. The 3D optical data are written in transparent glass by a computer-controlled 3D XYZ translation stage. A 465 nm light from xenon lamp is used to excite the glass, and the photochromism-induced luminescence modification is recorded by a CCD camera. The transmittance or luminescence modulation is recovered by the thermal stimulation.

The WO_3_ photochromic materials have been extensively exploited in amorphous films, powder, and polycrystalline ceramics^30^. The change of W^6+^ valence state to W^5+^ is responsible for the photochromism of WO_3_ materials^17,23^. In addition, the previous results have demonstrated that the phosphate glass is one of the most prospective photoluminescent matrixes for rare earth ions, which is mainly due to its high rare earth ion solubility, low nonlinear refractive index, large emission cross-section, etc^31^. In this study, the multifunctional tungsten phosphate glasses doped with rare earth ions were designed and prepared. The color of rare earth ions doped glass reversibly changes from light yellow to blue by alternating 473 nm laser illumination and thermal stimulation due to the change of W^6+^ valence state (Fig. 1), showing the writing and erasing ability. The reading out of optical information can be achieved by the transmittance and photoluminescence modulation of the rare earth doped glasses (Fig. 1). It is interesting that the optical information can be recorded in arbitrary 3D space of transparent glass, and the optical information written in the 3D space of transparent glass can be hierarchically discriminated, and thus the encryption function can be obtained.

## Results and discussion

Laser irradiation may induce defects in glass, which could bring photoluminescence^32,33^. No luminescence was observed in the pure tungsten phosphate glass before and after 473 nm laser irradiation (Fig. S1, Fig. S2), indicating the absence of intrinsic luminescence centers or irradiation-induced luminescence defects. The photoluminescence intensity of the Eu^3+^ doped tungsten phosphate glass showing a typical Eu^3+^ ions emission pattern is dependent on the concentration of Eu^3+^. Doping concentration of Eu^3+^ is determined to be 1 mol% in the tungsten phosphate glass (Fig. S1). The Sb_2_O_3_ addition has a significant influence on the transparency of the tungsten phosphate glasses. The glass without Sb_2_O_3_ is blue with low transparency. After adding Sb_2_O_3_, the glass exhibits high transparency in the region from 450 nm to 1800 nm (Fig. S3a). The glass with 1 mol% Sb_2_O_3_ exhibits the best transparency and strongest red luminescence (Fig. S3a, b). The poor transparency of pure glass without Sb_2_O_3_ is attributed to the hopping of polarons from W^6+^ to W^5+^ ^34,35^. The Sb_2_O_3_ addition stabilizes the valence state of W^6+^, preventing the transformation from W^6+^ to W^5+^ (Fig. S3c, d). Thus the transparent tungsten phosphate glasses are obtained. The glass with the molar composition of 50WO_3_-39.5NaH_2_PO_4_-8BaF_2_-0.5Na_2_CO_3_-1Sb_2_O_3_-1EuF_3_ was designed and prepared, which is denoted as WPG-Sb1.

**Fig. 2:**
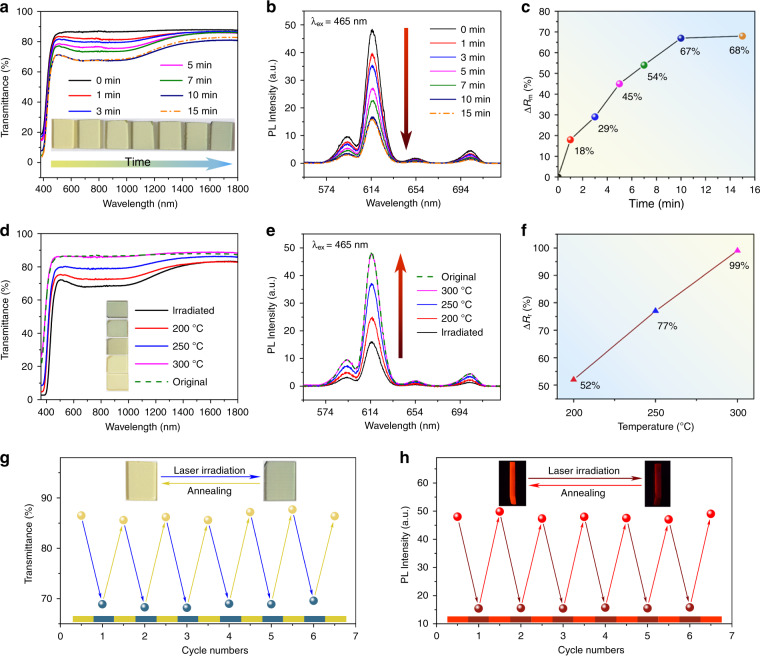
Reversible photochromism and luminescence modification. **a** Transmission spectra and photos of WPG-Sb1 glass irradiated by unfocused 473 nm laser (151.26 W cm^−2^) for various times. **b**, **c** Luminescence spectra (**b**) and corresponding modulation degree (**c**) of WPG-Sb1 glass scanned by unfocused 473 nm laser for various times under 465 nm excitation. **d** Transmission spectra and photos of blue WPG-Sb1 glass heat-treated at various temperatures for 10 min. **e**, **f** Luminescence spectra (**e**) and luminescence recovery degree (ΔR_r_) (**f**) of the blue WPG-Sb1 glass bleached by various temperatures for 10 min. **g**, **h** 614 nm transmittance (**g**) and luminescence intensity (**h**) of WPG-Sb1 glass by alternating 473 nm laser (151.26 W cm^−2^, 15 min) and thermal stimulation (300 °C, 10 min) as a function of cycle numbers.

The coloration of WPG-Sb1 glass was carried out under the scanning of the 473 nm laser, and the moving platform of the optical microscope was used to control the laser irradiation time on each point of the glass surface (Fig. 1). Figure 2a presents the transmission spectra and photos of WPG-Sb1 glass irradiated by the unfocused 473 nm laser (151.26 W cm^−2^) with a spot size of 1 mm for the various durations. The transmittance of the regions from 500 to 1400 nm of the glass starts to decrease when the laser irradiation time is about 1 min. With the increase of the irradiation time, the transmittance of the glass further decreases, and the blue color becomes deeper until achieving saturation after 10 min. The influence of 473 nm laser power density (irradiation time is 15 min) on the transmission spectra and photos of WPG-Sb1 glass is shown in Fig. S4a. At the same irradiation time, the transmittance decreases with the increase of the laser power density.

The photochromism reaction of WPG-Sb1 glass can be used to modulate its luminescence property. Figure 2b exhibits the luminescence spectra of WPG-Sb1 before and after the 473 nm laser (151.26 W cm^−2^) irradiation for various durations under the 465 nm excitation. As expected, the luminescence intensity of photochromic WPG-Sb1 glass gradually decreases with the irradiation time increase of 473 nm laser, and reaches stability at the irradiation time of 15 min. The luminescence modulation degree (Δ*R*_m_) of WPG-Sb1 glass is expressed by the Δ*R*_m_ = (*R*_0_ – *R*_i_)/*R*_0 _× 100%, where the *R*_0_ and *R*_i_ is the 614 nm initial luminescence intensity of raw WPG-Sb1 glass and the luminescence intensity of photochromic WPG-Sb1 glass treated by the 473 nm laser irradiation for various durations. The luminescence modulation degree increases with the increasing irradiation time of 473 nm laser (Fig. 2c). When the 473 nm irradiation times are 1, 3, 5, 7, 10, and 15 min, the calculated modulation degree of luminescence is 18%, 29%, 45%, 54%, 67% and 68%, respectively. At the optimized 15 min irradiation time, the influence of 473 nm laser power density on the luminescence modification is demonstrated in Fig. S4b. The increase of 473 nm laser power density results in the modulation degree (Δ*R*_m_) increase (Fig. S4c).

The bleaching of photochromic WPG-Sb1 glass irradiated by the 473 nm laser at a power density of 151.26 W cm^−2^ for 15 min was explored by the heat stimulation at various temperatures for different durations. Figure 2d exhibits the transmission spectra and photos of blue photochromic WPG-Sb1 glass heat-treated at various temperatures for 10 min. When the heat-treatment temperature is 200 °C, the blue color of the glass starts to fade. At 300 °C, the blue color has been completely faded. The photochromism-induced luminescence quenching could gradually be recovered when the blue WPG-Sb1 glass was heat-treated at various temperatures for 10 min (Fig. 2e). The recovery degree (Δ*R*_r_) of 614 nm luminescence is characterized by the Δ*R*_r_ = *R*_t_/*R*_0_*100%, where the *R*_t_ and *R*_0_ is the 614 nm luminescence intensity of the WPG-Sb1 photochromic glass heat-treated at various temperatures for 10 min and the 614 nm initial luminescence intensity of the raw WPG-Sb1 glass, respectively. The luminescence recovery degree increases with increasing the stimulation temperature (Fig. 2f). When the thermal stimulation temperature was 300 °C, the luminescence intensity of the glass was almost recovered to the corresponding original intensity.

The influence of thermal stimulation time at 200 °C on the decoloration and luminescence recovery of blue glass was investigated (Fig. S5). The transmittance and luminescence of photochromic WPG-Sb1 glass increase with the increase of stimulation time. When the heat-treatment time is 2 h, the color and luminescence of photochromic WPG-Sb1 glass can be recovered to the initial state, respectively. The reversible photochromism of WPG-Sb1 glass was observed upon the 473 nm laser and thermal stimulation, respectively. In order to study the reproducibility, the WPG-Sb1 glass was treated by alternating irradiation of 473 nm laser (151.26 W cm^−2^, 15 min) and thermal stimulus (300 °C, 10 min). As shown in Fig. 2g, the transmittance of the WPG-Sb1 glass can be switched off and on with excellent reproducibility after several cycles. The luminescence spectra of WPG-Sb1 upon alternating the 473 nm laser irradiation (151.26 W cm^−2^, 15 min) and thermal stimulus (300 °C, 10 min) have been measured as a function of the cycle numbers (Fig. 2h), exhibiting no deterioration of the luminescence. 50 cycles of transmittance and luminescence of the WPG-Sb1 glass were carried out (Fig. S6). It can be seen that degradation to the quality of the signal was not observed, and the excellent reversible property has been obtained. This glass with double reversible regulation of transmittance and luminescence may have potential applications in the fields of data storage and information encryption. As mentioned above, no luminescence from the glass host or defects are generated by the blue light irradiation in the tungsten phosphate glass. Therefore, the signal readout without interference can be obtained in the rare earth ions doped glass upon 465 nm excitation.

**Fig. 3:**
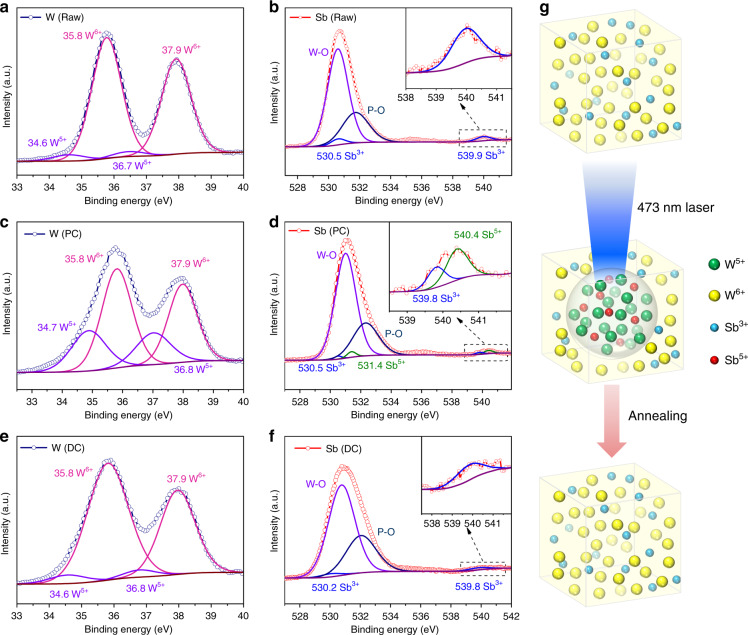
Reversible photochromic mechanism. **a**, **c** XPS spectra of W element in WPG-Sb1 before (**a**) and after (**c**) photochromism (PC) (151.26 W cm^−2^, 15 min). **b**, **d** XPS spectra of Sb element in WPG-Sb1 before (**b**) and after (**d**) photochromism (151.26 W cm^−2^, 15 min). **e**, **f** XPS spectra of W (**e**) and Sb (**f**) elements in the decolorated (DC) glass caused by heat-treatment at 300 °C for 10 min. **g** Proposed reversible photochromism mechanism of tungsten phosphate glass.

The reversible photochromism mechanism of the WPG-Sb1 glass was investigated by Raman, electron paramagnetic resonance (EPR), and X-ray photoelectron spectroscopy (XPS) spectra of the original, photochromic, or decolorated WPG-Sb1 glasses. The photochromism and decoloration of WPG-Sb1 glass were confirmed to be not attributed to the structural change of the glass (Fig. S7) or valence change of Eu^3+^ (Fig. S8). No peak of the oxygen vacancy was observed in the EPR spectra at room temperature of original, photochromic, and decolorated WPG-Sb1 glasses (Fig. S9), which suggests that the coloration of WPG-Sb1 glass is not attributed to the F-color center^36,37^. The grating structure and migration of elements were not observed in the photochromic region of WPG-Sb1 glass from the SEM image and energy-dispersive X-ray spectroscopy mapping (Fig. S10). The XPS spectra of W element in WPG-Sb1 before and after photochromism are shown in Fig. 3a, c. Two intensive 35.8 and 37.9 eV peaks of W^6+^ and two weak 34.6 and 36.7 eV peaks from W^5+^ were detected in the WPG-Sb1 glass before photochromism. It is interesting that the XPS peaks of W^5+^ significantly intensify in the photochromic WPG-Sb1 glass (151.26 W cm^−2^, 15 min). The results indicate that 473 nm laser irradiation induces the transformation from W^6+^ to W^5+^. XPS spectra of Sb element in WPG-Sb1 before and after photochromism are shown in Fig. 3b, d. 539.9 and 530.5 eV binding energies of Sb^3+^ were detected in the XPS spectra, and no XPS peak of Sb^5+^ were observed in WPG-Sb1 before photochromism. While the typical XPS peaks of Sb^5+^ can be observed in the photochromic WPG-Sb1, which locate at 531.4 and 540.4 eV^38,39^. The following reactions Sb^3+ ^+ *hv* → Sb^5+^ + 2e^−^ and W^6+^ + *hv* + e^−^ → W^5+^ may happen for the WPG-Sb1 glass upon 473 nm laser irradiation. The hopping of polarons from W^6+^ to W^5+^ is responsible for the photochromism of WPG-Sb1 glass due to the W^5+^ generation^34^.

Other light sources such as 532 (284.19 W cm^−2^), 808 (78.53 W cm^−2^), and 980 nm (53.47 W cm^−2^) lasers were used to irradiate the WPG-Sb1 glass. We demonstrated that the WPG-Sb1 glass also exhibits the photochromic property upon 532 nm laser stimulation (Fig. S11), while no photochromism is observed upon 808 and 980 nm lasers stimulation. The photochromic effect of the WPG-Sb1 glass upon 473 nm laser irradiation is better than that by 532 nm laser irradiation at the same irradiation time (Fig. S11). Sb^3+^ exhibits a broad absorption ranging from 350 nm to 700 nm with a peak at 480 nm^40^. The reaction between Sb^3+^ and W^6+^ in the WPG-Sb1 glass may be related to the absorption of Sb^3+^ upon 473 or 532 laser irradiation. The WPG-Sb1 glass irradiated for 1 h by 465 nm light of xenon lamp exhibits no photochromism due to lower power density (Fig. S11). XPS spectra of decolorated WPG-Sb1 glass after heat-treatment at 300 °C for 10 min were measured. As shown in Fig. 3e, f, the XPS peak intensities of W^6+^ and Sb^3+^ in the decolorated WPG-Sb1 glass increase in comparison with those in the photochromism sample. By contrast, the XPS peak intensity of W^5+^ decreases in the decolorated WPG-Sb1 glass after heat-treatment, and the XPS peak of Sb^5+^ disappears. The W^5+^ → W^6+^ + e^−^ and Sb^5+ ^+ 2e^− ^→ Sb^3+^ reactions take place in the photochromic WPG-Sb1 glass upon thermal stimulation. The thermal stimulation results in the decrease of W^5+^ amount in the photochromic WPG-Sb1 glass, causing its decoloration.

The luminescence of WPG-Sb1 has been successfully modulated by its photo-control transmittance. The mechanisms of radiative energy transfer and resonance energy transfer can be used to explain this luminescence modulation^41,42^. The radiative energy transfer and resonance energy transfer can be characterized by the decay lifetime of active centers. Figure S12 presents the decay curves of 614 nm emission of the glass upon 473 nm irradiation for different durations. The decay lifetime of 614 nm emission from Eu^3+^ slightly decreases with the increase of the irradiation time, which suggests that the luminescence modulation is not mainly attributed to the resonance energy transfer. The luminescence mechanism is presented in Fig. S13a. The radiative transitions from ^5^D_0_ to ^5^F_n_ (*n* = 1, 2, 3, and 4) of Eu^3+^produces the 593, 614, 650, and 700 nm luminescence, respectively. The luminescence of Eu^3+^ overlaps with the absorbance of the photochromic WPG-Sb1 glass (Fig. S13b). Thus the luminescence of Eu^3+^ can be absorbed by the blue glass host, resulting in the luminescence modulation.

**Fig. 4:**
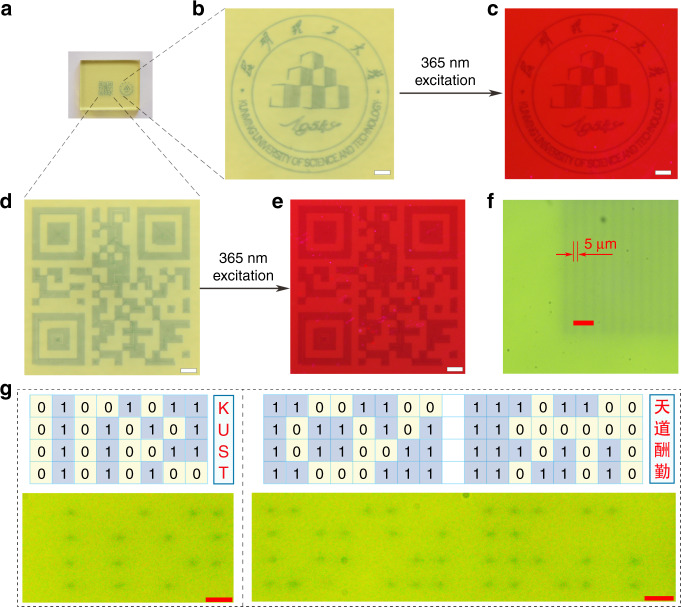
Optical information writing and readout. **a**, **b**, **d** The raw (**a**) and enlarged (**b**, **d**) photochromic logo patterns. **c**, **e** The enlarged luminescent logo pattern upon the 365 nm excitation. **d**, **e** The information data were hidden into the photochromic (**d**) or luminescent (**e**) two-dimensional code pattern, and the information “I love KUST” can be obtained by scanning the two-dimensional code pattern. Scale bar: 500 μm. **f** The scanning line width of focused 473 lasers. Scale bar: 40 μm. **g** The “KUST” alphabet (left) and the “tian dao chou qin” idiom (right) recorded into the transparent glass by binary format. Scale bar: 20 μm.

In order to demonstrate the optical storage application of tungsten phosphate glasses, the as-prepared WPG-Sb1 glass was irradiated by the focused 473 nm laser. The used power density of focused 473 nm laser is about 1915 kW cm^−2^, below the 2058 kW cm^−2^ power density threshold of glass damage (Fig. S14). The single scanning line width and spot size of focused 473 lasers are about 5 μm (Fig. 4f, g), and the resolution can be improved by the laser spot reduction. The laser irradiation time was shortened to 0.02 s to get the saturated photochromic WPG-Sb1 glass because the 473 nm laser focusing caused power density improvement. Photochromic complicated logo pattern was written at the surface of WPG-Sb1 glass by the focused laser direct writing technology (Fig. 4a, b). Based on the luminescence modification induced by the photochromism reaction, the luminescence logo pattern at the surface of WPG-Sb1 glass could also be revealed by using 365 nm UV lamp (Fig. 4c). It is interesting that the information data can be stored in the photochromic or luminescent two-dimensional code pattern (Fig. 4d, e). The reading out of information could be achieved by using intelligent mobile phone to scan the two-dimensional code pattern, as exhibited by the information “I love KUST” (Supplementary Video 1). The photochromic pattern can be obviously observed after ten months (Fig. S15), which suggests the photochromic glass has excellent chemical stability. Reading out of information can be obtained by the change of transmittance or luminescent intensity, which corresponds to “0” and “1” state in the binary system, respectively (Fig. 2g). The binary dot arrays encoded by common computer file formats (Fig. S16) were written into the transparent glass, for example to generate the binary data of “KUST” alphabet and the Chinese idiom of “tian dao chou qin” (Fig. 4g). The photochromic or luminescent pattern can be erased by the thermal stimulation, and the photochromic reversibility of the glass ensures its reusing (Fig. S17). In addition, other rare earth ions (such as Dy^3+^) doped glass with the composition of 50WO_3_-39.5NaH_2_PO_4_-8BaF_2_-0.5Na_2_CO_3_-1Sb_2_O_3_-1DyF_3_ was prepared. The blue photochromism and yellow luminescence patterns of Dy^3+^ were obtained (Fig. S18), demonstrating the universality of the technology.

**Fig. 5:**
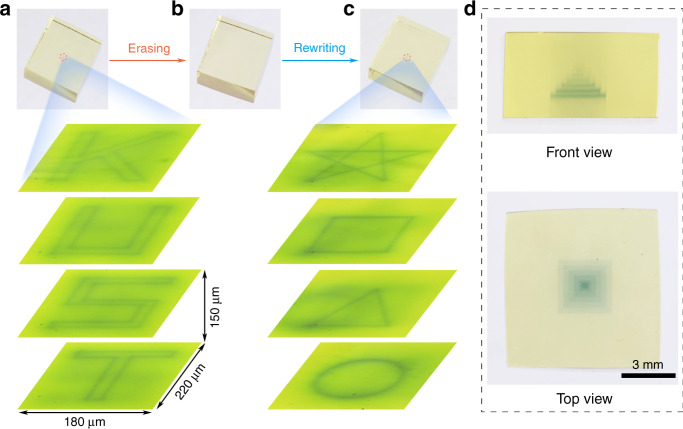
Demonstration of 3D optical data storage and information encryption. **a** The 3D optical information written in various layers of the glass, the photograph of the transparent glass with the “KUST” information under the daylight (upper photo), and the optical microscope images of “KUST” information observed by using an optical microscope. **b** The photograph of glass after the “KUST” information erasing. **c** The 3D optical information rewritten in various layers of the glass, the photograph of the transparent glass with the “pentacle, quadrate, triangle and circle pattern” information under daylight (upper photo) and the optical microscope images of “pentacle, quadrate, triangle and circle” information observed by using optical microscope. **d** The 3D structure inside the glass from the front view and top view under daylight.

The inorganic photochromic glass exhibits many advantages such as the ability of 3D optical data storage due to its transparent property. As shown in Fig. 5a, the 3D optical information was written in various layers of the glass by the 473 nm laser direct writing technology, and the “KUST” four letters in the different layers can be observed by an optical microscope. The 3D optical data stored in the glass can be erased by the thermal stimulation (Fig. 5b). The “pentacle, quadrate, triangle, and circle” optical information was rewritten in the different layers of the glass (Fig. 5c), exhibiting the reversible 3D optical data storage ability of the glass. It is noted that the complicated 3D structure can be written inside the glass by the 473 nm direct laser writing technology (Fig. 5d), exhibiting the universality of the technology. In addition, the photo-modulated glass not only shows the ability of the reversible 3D optical data storage, but also opens a new opportunity in the information encryption. The “KUST” optical information written in various layers of the transparent glass can be hierarchically discriminated using an optical microscope (Supplementary Video 2), and thus the encryption function can be obtained.

In summary, the photochromism of rare earth ions doped tungsten phosphate transparent glass was demonstrated under the stimulation of 473 nm laser, which could be tailored by the laser power density or laser irradiation time. The decoloration of photochromic glass was obtained by thermal stimulation. The photochromism of rare earth ions doped tungsten phosphate transparent glass exhibited excellent reversibility, reproducibility and stability. The photoluminescence intensity was reversibly modified by the transmittance modulation of the rare earth ions doped glasses. The complex information patterns can be written and erased in the photo-modulated glass, showing the ability of the reversible 3D optical data storage. In addition, the optical information written in the arbitrary 3D space of transparent glass can be hierarchically discriminated, demonstrating the information encryption function. We believe that this photo-modulated glass is of importance for extending its new applications in the optoelectronic fields.

## Materials and methods

### Glass preparation

WO_3_ (99.99%), BaF_2_ (99.99%), Sb_2_O_3_ (99.99%), Na_2_CO_3_(99.99%), EuF_3_ (99.99%), DyF_3_ (99.99%) and NaH_2_PO_4_ (99.0%) were used as raw materials for the preparation of tungsten phosphate glasses doped with Eu^3+^ or Dy^3+^. Tungsten phosphate glasses with the molar compositions of (51-*x*)WO_3_-39.5NaH_2_PO_4_-8BaF_2_-0.5Na_2_CO_3_-*x*Sb_2_O_3_-1EuF_3_ (*x* = 0, 0.5, 1 and 2) were prepared by the conventional melt-quenching method. The raw materials were mixed homogeneously in an agate mortar, and then were melted at 1050 °C for 1 h in air. Then the melted tungsten phosphate glass was poured onto a brass mold and cooled down to room temperature. The obtained glass was annealed at 420 °C for 5 h to remove thermal strains. Then polished glass with appropriate size was obtained before the laser irradiation. To investigate the influence of Eu^3+^ concentration on the luminescence, the 50WO_3_-(40.5-*y*) NaH_2_PO_4_-8BaF_2_-0.5Na_2_CO_3_-1Sb_2_O_3_-*y*EuF_3_ (*y* = 0, 0.25, 0.5, 1.0, 1.5 and 2.0) tungsten phosphate glasses were prepared by the same approach.

### Reversible writing and erasing of optical information in the glass

The coloration of glass was carried out under the irradiation of 473 nm laser with and without focusing. The 473 nm laser (MBL-N-473 nm-1 W) was focused by a 50× (NA = 0.55; WD = 8.2 mm) objective lens (EPLE-50). The resolution of the laser spot was about 5 μm. The focusing laser power density (1915 kW cm^-2^) is slightly below the threshold power density of glass damage. The optical shutter (SR470) was used to control the on/off of laser irradiation. The moving platform of the optical microscope (Axio Imager A1m) was used to control the laser irradiation time by using the computer software program to manipulate the movement speed of the glass. The optical data were erased by thermal stimulus at different temperatures and durations.

### Sample characterization

The optical microscope images were captured by a Nikon (AZ100M) optical microscope equipped with a CCD camera. The photochromic images under daylight and photoluminescence images under the excitation of 365 nm UV lamp were taken by a CCD camera (Nikon D7100, Japan). A mobile phone with a commercial APP official QR code Reader was used for QR code recognition (Supplementary video 1). The transmission spectra of tungsten phosphate glass samples before and after photochromism were measured by the U-4100 spectrophotometer. The luminescence and excitation spectra of tungsten phosphate glass were measured by the HITACHIU-F-7000 spectrophotometer using Xe lamp as the light source. An Edinburgh FLS 980 instrument (Edinburgh Instruments Ltd., Livingston, UK) was used to measure the lifetime decay of the glasses. The chemical states of tungsten phosphate glass were characterized by XPS (200 W) with Al Kα radiation (Thermo Fisher Scientific) under vacuum conditions. For the Raman spectra measurement of the samples, the Argon laser with continuous wave (*λ* = 514 nm) was used as the excitation source. The Bruker model ELEXSYS-IIE500 spectrometer (Bruker, Switzerland) was used to measure the EPR spectra at room temperature. The element distribution was detected by a Zeiss sigma 300 scanning electron microscope with an energy-dispersive X-ray spectrometer (SmartEDX).

## Supplementary information

Supporting information

The reading out of information

Multi-level information encryption
