# Supplementary material for: Reversible 3D optical data storage and information encryption in photo-modulated transparent glass medium
Source: Light Sci Appl. 2021 Jul 7;10:140. doi: 10.1038/s41377-021-00581-y (PMC8263721; doi:10.1038/s41377-021-00581-y)
Supplement: Supplementary file 1 — Supporting information [file 41377_2021_581_MOESM1_ESM.pdf]

Supplementary information

Reversible 3D optical data storage and information encryption in photo-modulated transparent glass medium

Zhen Hu[1], Xiongjian Huang[2], Zhengwen Yang[1]*, Jianbei Qiu[1], Zhiguo Song[1], Junying Zhang[3]*, Guoping Dong[2]*

[1]College of Materials Science and Engineering, Kunming University of Science and Technology, 650093, Kunming, China.
[2]State Key Laboratory of Luminescent Materials and Devices, School of Materials Science and Engineering, South China University of Technology, 510640, Guangzhou, China.
[3]School of Physics, Beihang University, 100191, Beijing, China.
Corresponding Author e-mails: yangzw@kust.edu.cn; zjy@buaa.edu.cn; dgp@scut.edu.cn

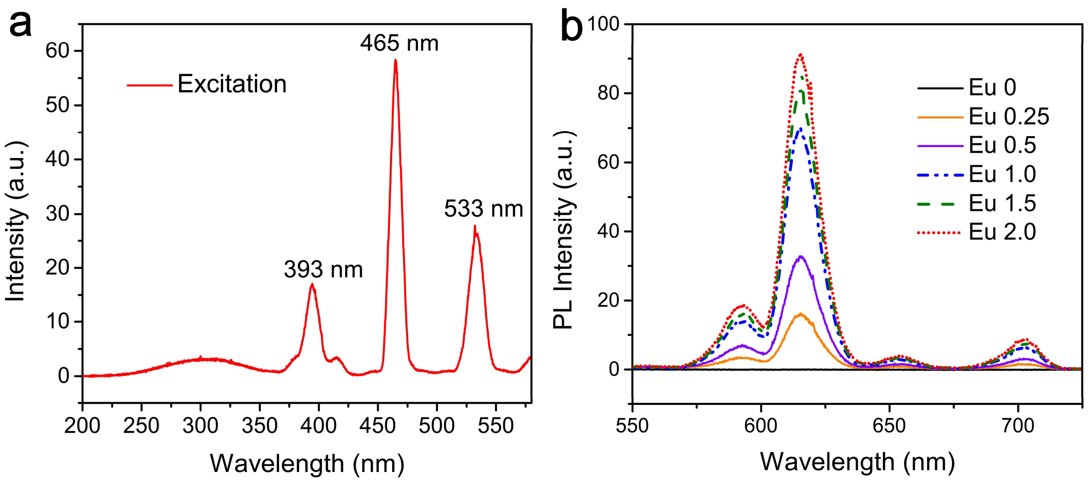

Fig. S1. a, Excitation spectrum by monitoring 614 nm emission of glass with the molar composition of $50WO_3$-$39.5NaH_2PO_4$-$8BaF_2$-$0.5Na_2CO_3$-$1Sb_2O_3$-$1EuF_3$. **b,** Luminescence spectra of glasses with the molar compositions of $50WO_3$-$(40.5\text{-}y)NaH_2PO_4$-$8BaF_2$-$0.5Na_2CO_3$-$1Sb_2O_3$-$yEuF_3$ (y=0, 0.25, 0.5, 1.0, 1.5 and 2.0) under the excitation of 465 nm.

Figure S1a exhibits the excitation spectrum of glasses with the molar composition of $50WO_3$-$39.5NaH_2PO_4$-$8BaF_2$-$0.5Na_2CO_3$-$1Sb_2O_3$-$1EuF_3$. The three excitation peaks of Eu^{3+} can be observed by monitoring 614 nm emission, which are located at 393, 465 and 533 nm. Under the excitation of 465 nm, the glass without Eu^{3+} has no luminescence. The typical luminescence peaks at 593, 614, 650 and 700 nm of Eu^{3+} were observed in the glasses with the molar compositions of $50WO_3$-$(40.5\text{-}y)NaH_2PO_4$-$8BaF_2$-$0.5Na_2CO_3$-$1Sb_2O_3$-$yEuF_3$ (y=0, 0.25, 0.5, 1.0, 1.5 and 2.0), as shown in Fig. S1b. The luminescence significantly increases with increasing Eu^{3+} concentration from 0.25 to 1 mol%, and then slowly increases with increasing Eu^{3+} concentration from 1 to 2 mol%. Doping concentration of Eu^{3+} in the glass was selected as 1 mol% in the following research.

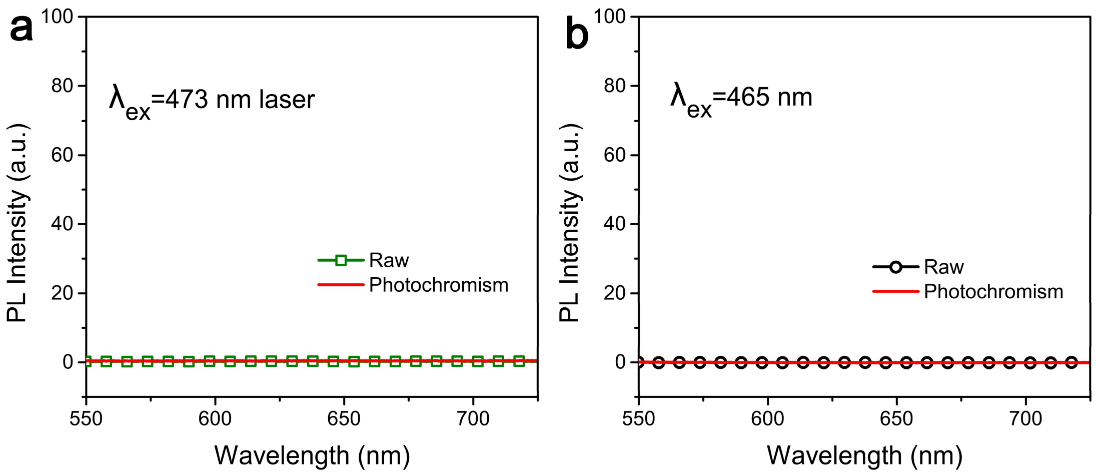

Fig. S2. a, b, Luminescence spectra of glasses with molar composition of $50WO_3$-$40.5NaH_2PO_4$-$8BaF_2$-$0.5Na_2CO_3$-$1Sb_2O_3$ without Eu^{3+} doping before and after photochromism of 473 nm laser. (a) the excitation wavelength for luminescence measurement is 473 nm laser, (b) the excitation wavelength for luminescence measurement is 465 nm light from xenon lamp.

Figure S2 exhibits the luminescence spectra of glasses without Eu^{3+} doping. It is shown that the defect luminescent centers are not generated by intense blue irradiation in the tungsten phosphate glass. Therefore, the fluorescence signal of glass with rare earth ions will not be influenced after intense blue irradiation.

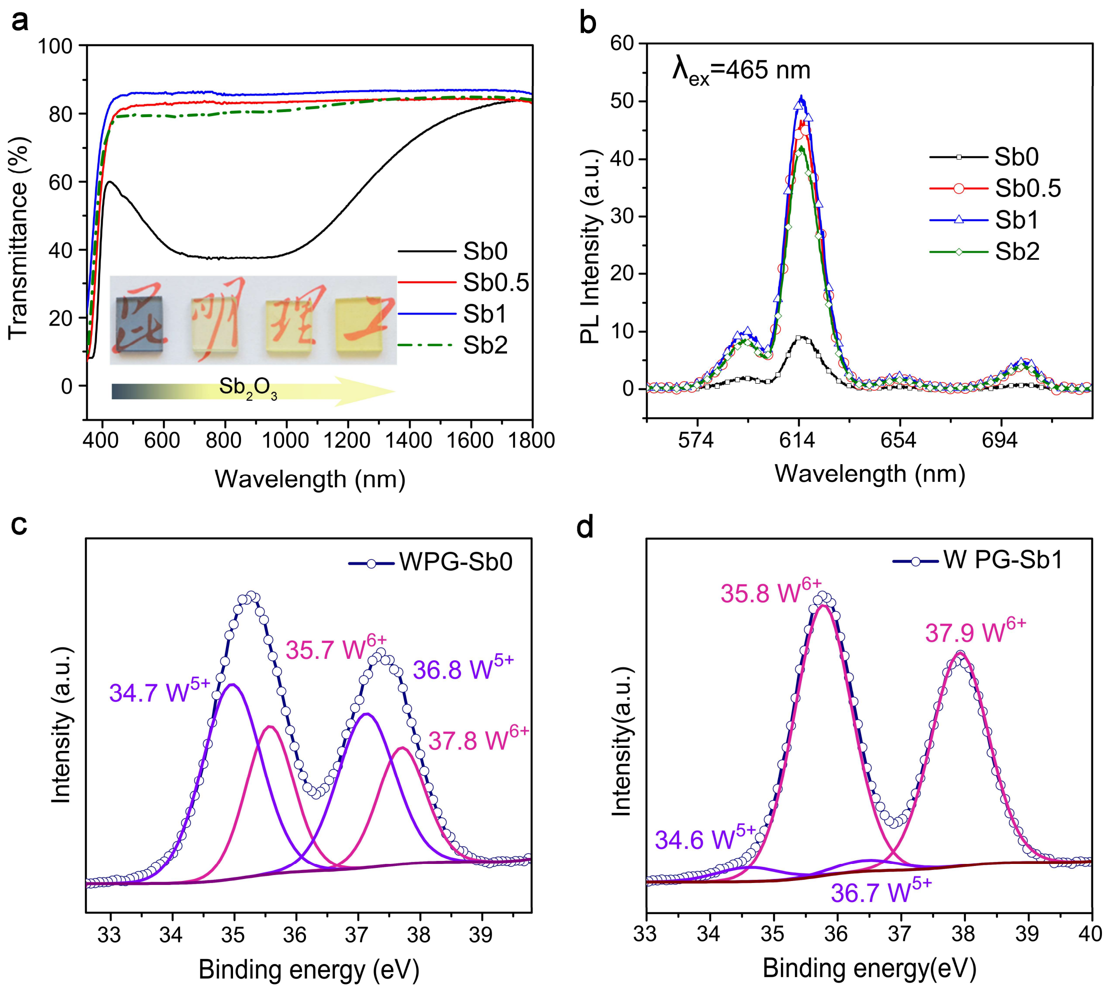

Fig. S3. a, Transmission spectra and photos of the glasses with the molar compositions of (51-x)WO_3-39.5NaH_2PO_4-8BaF_2-0.5Na_2CO_3-xSb_2O_3-1EuF_3 (x=0, 0.5, 1 and 2). **b,** Luminescence spectra of the (51-x)WO_3-39.5NaH_2PO_4-8BaF_2-0.5Na_2CO_3-xSb_2O_3-1EuF_3 (x=0, 0.5, 1 and 2) glasses under 465 nm excitation. **c, d,** XPS spectra of W element in the glass without (**c**) and with (**d**) the addition of 1 mol% Sb_2O_3.

The glasses with the molar compositions of (51-x)WO_3-39.5NaH_2PO_4-8BaF_2-0.5Na_2CO_3-xSb_2O_3-1EuF_3 (x=0, 0.5, 1 and 2 mol%) were designed and prepared. As shown in Fig. S3a, the Sb_2O_3 addition has significant influence on the transparency of the tungsten phosphate glasses. The glass without Sb_2O_3

addition is blue. While the glasses including 0.5 or 1 mol% Sb_2O_3 exhibit the excellent transparency in the region from 450 nm to 1800 nm in contrast to the glass including 2 mol% Sb_2O_3, and the optimum concentration of Sb_2O_3 is 1 mol%. Figure S3b exhibits the luminescence spectra of glasses with various concentrations of Sb_2O_3 upon 465 nm excitation. The four luminescence peaks of Eu^{3+} were observed, locating at 593, 614, 650 and 700 nm. Compared with the glasses with Sb_2O_3, the glass without Sb_2O_3 exhibits low luminescence intensity. The luminescence of Eu^{3+} overlaps with the absorbance of the glass without Sb_2O_3. Thus the luminescence of Eu^{3+} can be absorbed by the blue glass host, resulting in luminescence decreasing. The blue color of glass without Sb_2O_3 is attributed to the hopping of polarons from W^{6+} to W^{5+}. Two 35.7 and 37.8 eV XPS peaks of W^{6+} have been observed, and the intensive 34.7 and 36.8 eV XPS peaks are from W^{5+} in the blue glass without Sb_2O_3 (Fig. S3c). It is obvious that the XPS peaks of W^{5+} significantly decrease in the glasses after the addition of 1 mol% Sb_2O_3 (Fig. S3d). The Sb_2O_3 addition stabilizes the valence state of W^{6+}, preventing the transformation from W^{6+} to W^{5+}, and thus the transparent tungsten phosphate glasses were obtained.

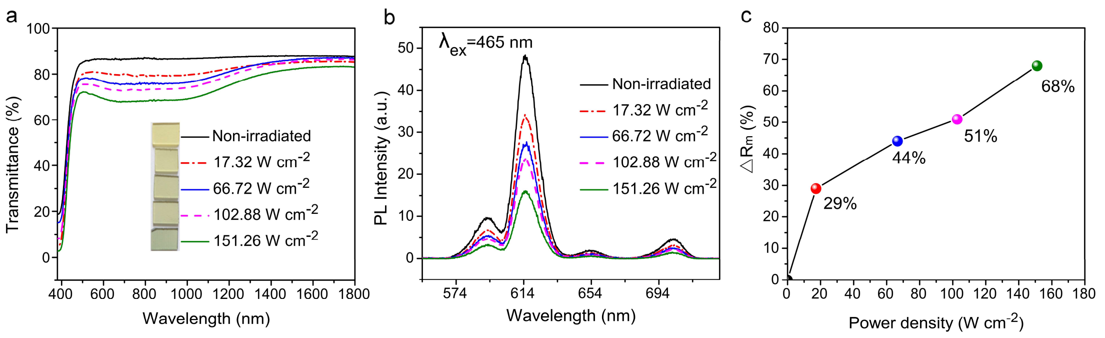

Fig. S4. a, Transmission spectra and photos of WPG-Sb1 glass scanned by unfocused 473 nm laser (spot size is 1 mm) with various power density for 15 min. **b,** Luminescence spectra of WPG-Sb1 before and after 473 nm laser irradiation with various power densities for 15 min upon 465 nm excitation. **c,** Modulation degree (ΔR_m) of luminescence as a function of laser power density.

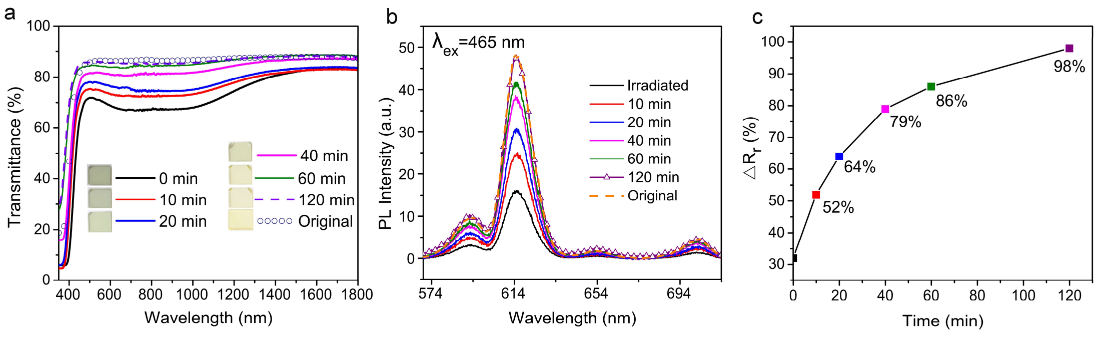

Fig. S5. a, Transmission spectra and photos of blue WPG-Sb1 heat-treated at 200 °C for various durations. **b, c,** Luminescence spectra (**b**) and recovery degree (ΔR_r) (**c**) of the glass bleached by 200 °C heat-treatment for various durations.

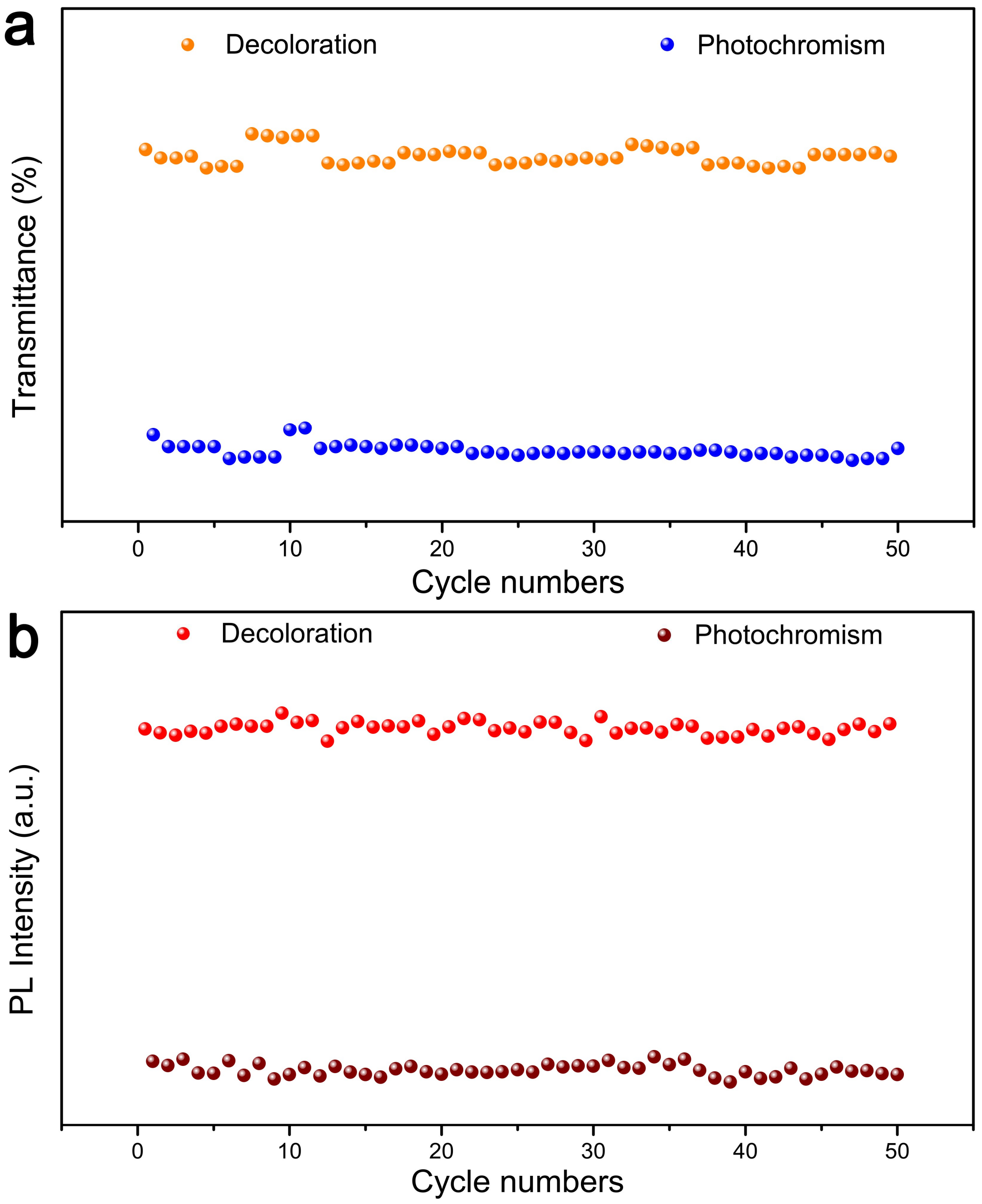

Fig. S6. 614 nm transmission (**a**) and luminescence intensity (**b**) of WPG-Sb1 glass by alternating 473 nm laser (151.26 W cm^{-2}, 15 min) and thermal stimulation (300 °C, 10 min) for 50 cycles.

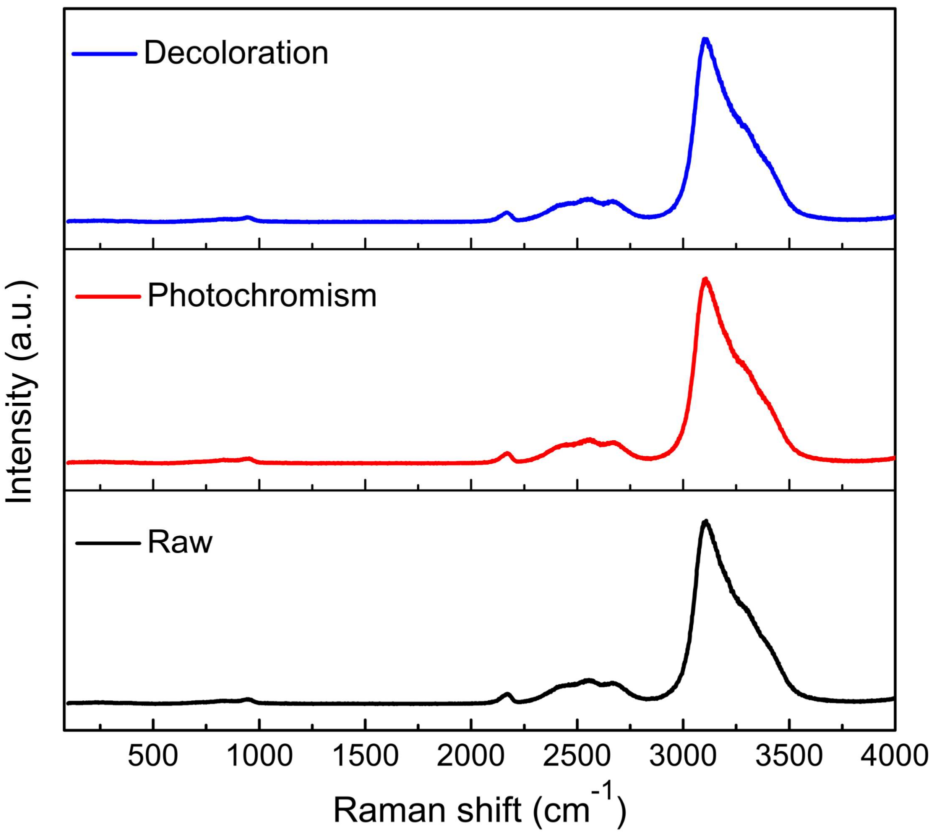

Fig. S7. Raman spectra of raw, photochromic and decolorated WPG-Sb1 glasses.

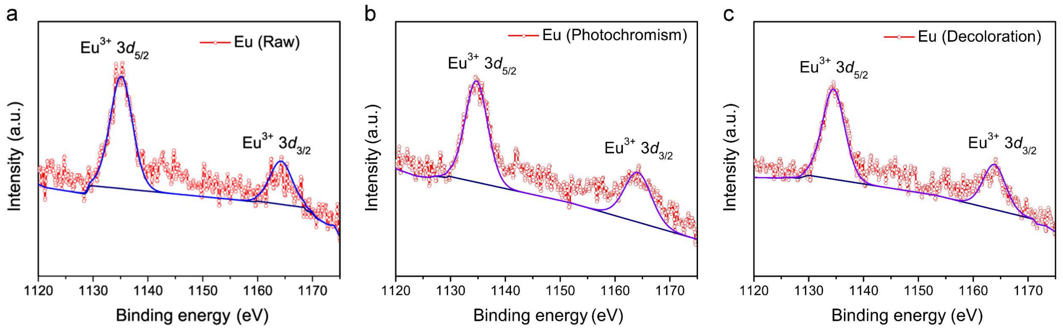

Fig. S8. XPS spectra of Eu^{3+} element in the raw, photochromic and decolorated WPG-Sb1 glasses.

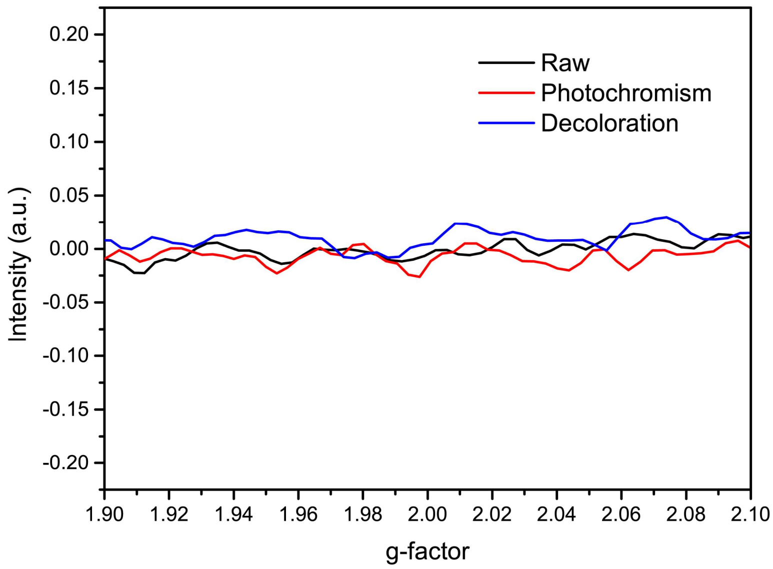

Fig. S9. EPR spectra at room temperature of the raw, photochromic and decolorated WPG-Sb1 glasses.

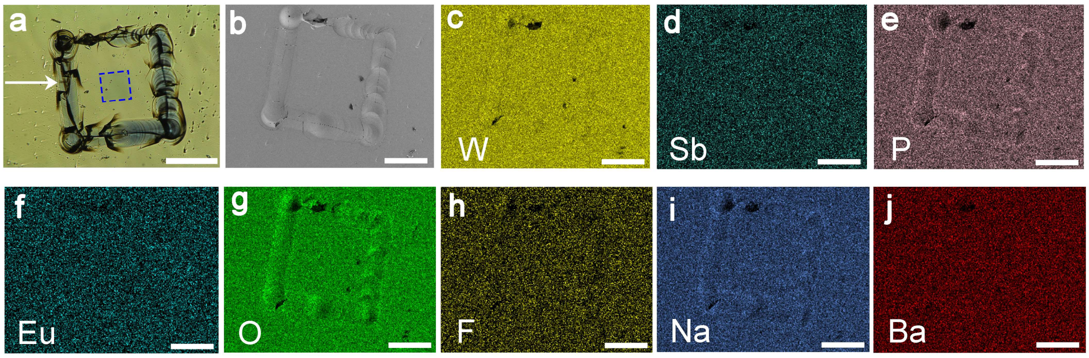

Fig. S10. a, Optical microscopic image of photochromism in the WPG-Sb1 glass. The big rectangular identified by arrows was obtained by the irradiation of focused 473 nm laser (2058 kW cm^{-2}). The rectangle blue region is the position of the irradiated regions of unfocused 473 nm laser (151.26 W cm^{-2}). **b,** SEM image corresponding to **a**. **c-j,** Corresponding EDS elemental mapping images. Scale bar, 200 μm. No obvious element-migration can be observed in the photochromic regions.

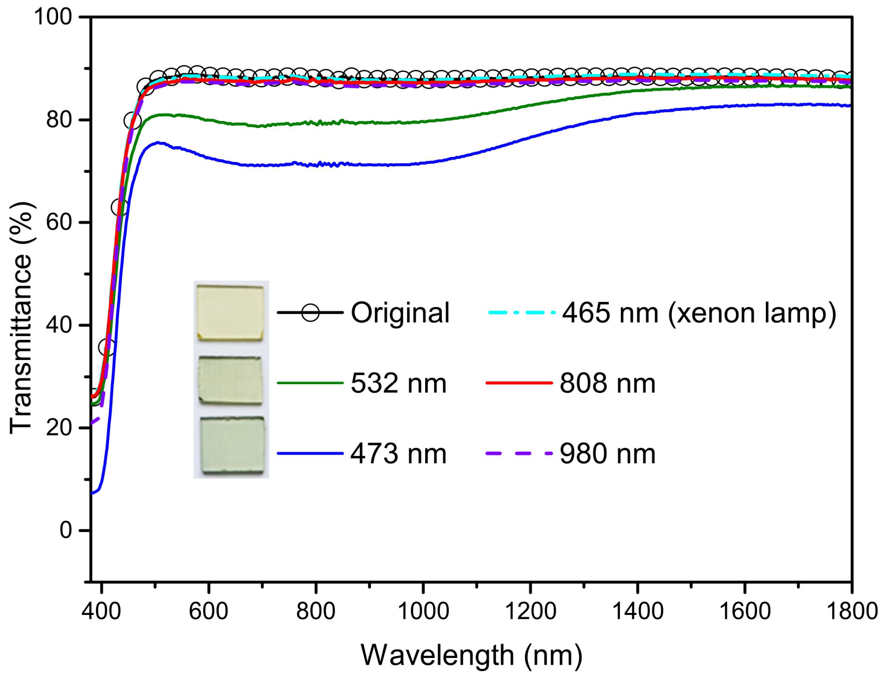

Fig. S11. Transmission spectra and photos of WPG-Sb1 glass under irradiation of 473 nm (151.26 W cm^{-2}) and 532 nm (284.19 W cm^{-2}) laser for 15 min, and 808 nm (78.53 W cm^{-2}), 980 nm (53.47 W cm^{-2}) lasers and 465 nm xenon lamp for 1 h.

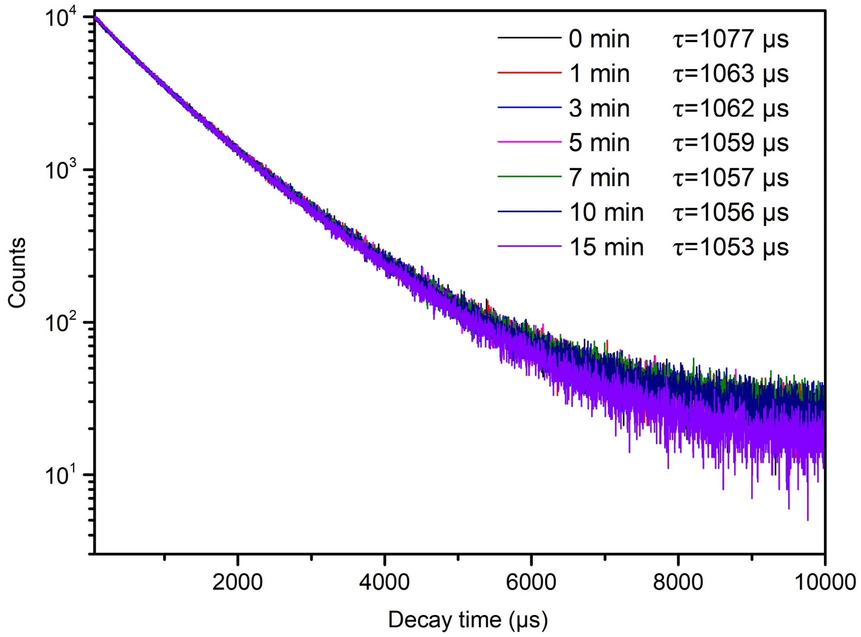

Fig. S12. Decay curves of 614 nm luminescence of the glass irradiated by 473 nm laser (151.26 W cm^{-2}) for various durations.

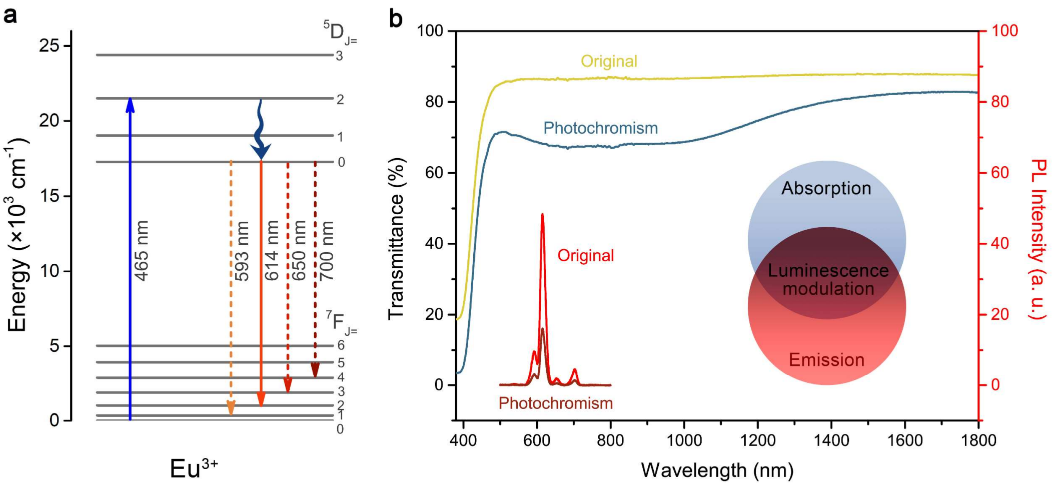

Fig. S13. **a,** Luminescence mechanism of Eu^{3+} in the WPG-Sb1 glass. **b,** Luminescence modification mechanism of Eu^{3+}.

The $^5D_2$ energy level of Eu^{3+} was populated upon 465 nm excitation, as shown in Fig. S13a. The electrons relaxed to the $^5D_0$ energy level. The radiative transitions from $^5D_0$ to ^5F_n (n=1, 2, 3, 4) of Eu^{3+} produce luminescence peaking at 593, 614, 650 and 700 nm, respectively. The luminescence of Eu^{3+} overlaps with the absorbance of the photochromic glass, as shown in Fig. S13b. Thus the luminescence of Eu^{3+} can be absorbed by the blue glass host, resulting in the luminescence modulation.

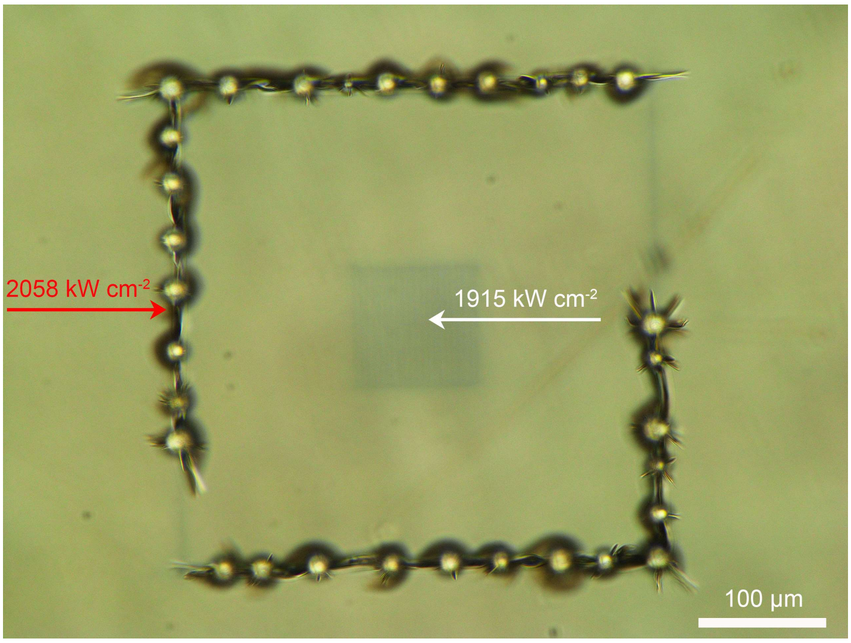

Fig. S14. 473 nm laser was focused by a 50× (NA=0.55; WD=8.2 mm) objective lens (EPLE-50) on the glass. Damage of the glass was observed when the power density (2058 kW cm^{-2}) of focused 473 nm laser is slightly greater than the power density for writing (1915 kW cm^{-2}).

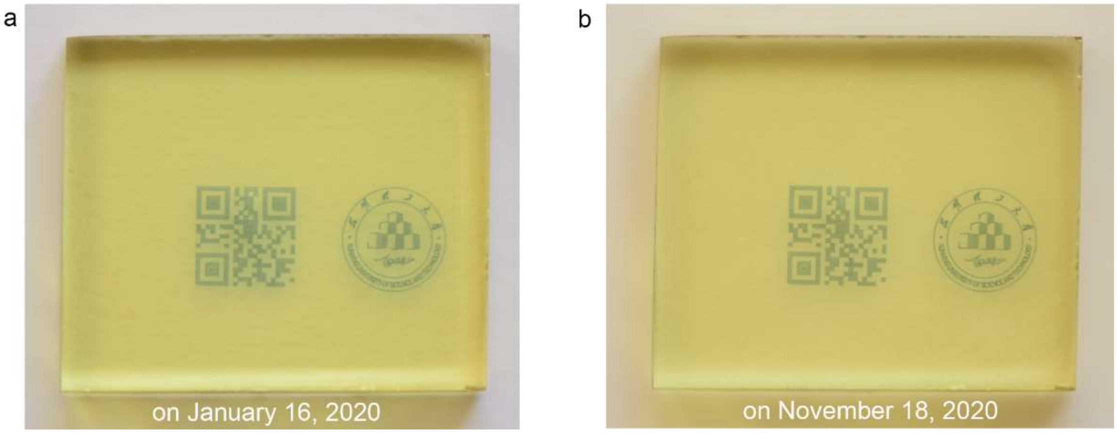

Fig. S15. Information was chronically stored under natural environment in the transparent glass. **a,** photo taken on January 16, 2020. **b,** photo taken on November 18, 2020 of the same sample in (**a**).

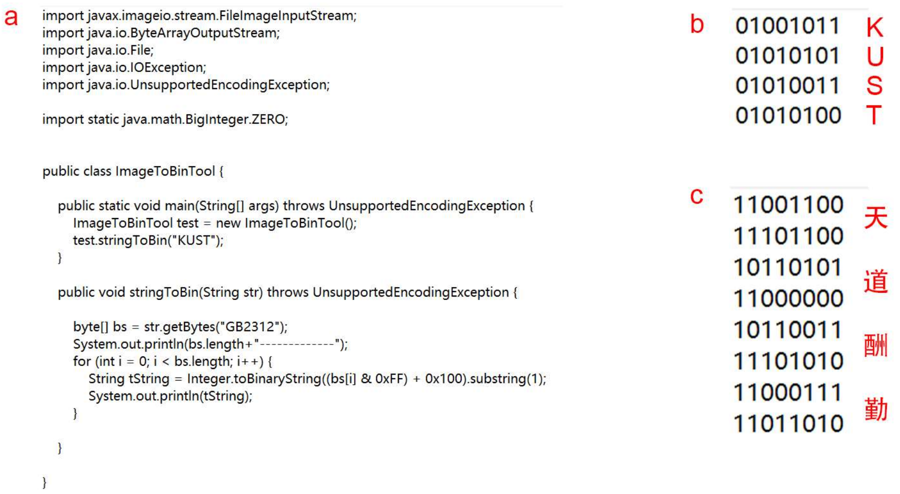

Fig. S16. **a,** Information can be encoded to binary data by common computer programming file. **b, c,** Binary data of a “KUST” alphabet (**b**) and the Chinese idiom “tian dao chou qin” (**c**).

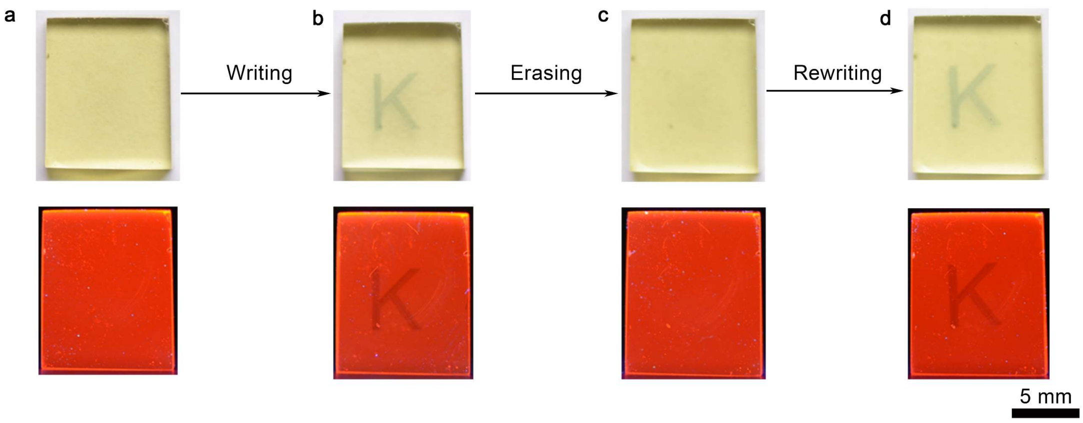

Fig. S17. Erasing and recovering photochromic and luminescent patterns by alternating focused 473 nm laser (1915 kW cm^{-2}, 250 μm s^{-1}) and thermal stimulation (300 °C, 10 min).

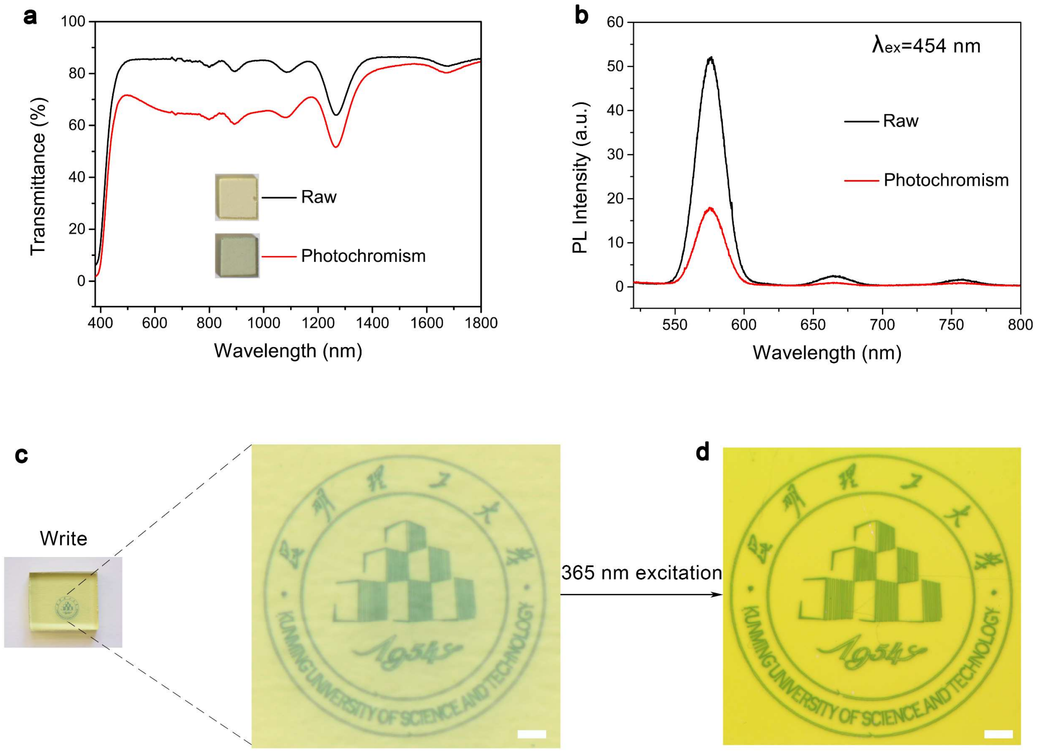

Fig. S18. **a,** Transmission spectra and photos of the $50WO_3$-$39.5NaH_2PO_4$-$8BaF_2$-$0.5Na_2CO_3$-$1Sb_2O_3$-$1DyF_3$ glass scanned by unfocused 473 nm laser (151.26 W cm^{-2}) for 15 min. **b,** Luminescence spectra of the $50WO_3$-$39.5NaH_2PO_4$-$8BaF_2$-$0.5Na_2CO_3$-$1Sb_2O_3$-$1DyF_3$ glass before and after photochromism. **c, d,** The photochromic (**c**) and photoluminescent (**d**) logo patterns of the $50WO_3$-$39.5NaH_2PO_4$-$8BaF_2$-$0.5Na_2CO_3$-$1Sb_2O_3$-$1DyF_3$ glass by the focused 473 nm laser direct writing (1915 kW cm^{-2}). Scale bars, 500 μm.
